# The Impact of Time Interval between Hepatic Resection and Liver Transplantation on Clinical Outcome in Patients with Hepatocellular Carcinoma

**DOI:** 10.3390/cancers13102398

**Published:** 2021-05-15

**Authors:** Matteo Serenari, Enrico Prosperi, Marc-Antoine Allard, Michele Paterno, Nicolas Golse, Andrea Laurenzi, René Adam, Matteo Ravaioli, Daniel Cherqui, Matteo Cescon

**Affiliations:** 1 General Surgery and Transplant Unit, IRCCS, Azienda Ospedaliero-Universitaria di Bologna, Sant’Orsola-Malpighi Hospital, 40138 Bologna, Italy; matteo.serenari@aosp.bo.it (M.S.); andrea.laurenzi@aosp.bo.it (A.L.); matteo.ravaioli@aosp.bo.it (M.R.); 2 Department of Medical and Surgical Sciences, Alma Mater Studiorum, University of Bologna, 40138 Bologna, Italy; enrico.prosperi@studio.unibo.it; 3 Centre Hépato-Biliaire, AP-HP Hôpital Paul Brousse, 94805 Villejuif, France; marcantoine.allard@aphp.fr (M.-A.A.); nicolas.golse@aphp.fr (N.G.); rene.adam@aphp.fr (R.A.); daniel.cherqui@aphp.fr (D.C.); 4 School of General Surgery, University of Milan, 20157 Milan, Italy; michele.paterno1@unimi.it

**Keywords:** liver transplantation, hepatectomy, outcome, benchmark, hepatocellular carcinoma

## Abstract

**Simple Summary:**

Recurrence of disease or worsening of liver function after hepatic resection (HR) for hepatocellular carcinoma (HCC) may require secondary liver transplantation (SLT). However, a history of HR is supposed to increase the surgical complexity of LT. This is one of the largest series of SLT and it demonstrates that among all the features analyzed regarding the prior HR, only time interval between HR and SLT was an independent predictor of severe complications after SLT. In particular, an increasing probability of severe complications was observed in those patients who were transplanted close (<15 months) to the HR. There was no significant association between HCC-related death and the time between HR and SLT at the multivariable competing risks regression model. Furthermore, these results remained inside the benchmark values recently reported for LT, confirming that tertiary referral centers with consistent experience in HPB surgery and LT may have benefits in both fields.

**Abstract:**

Hepatic resection (HR) for hepatocellular carcinoma (HCC) may require secondary liver transplantation (SLT). However, a previous HR is supposed to worsen post-SLT outcomes. Data of patients treated by SLT between 2000 and 2018 at two tertiary referral centers were analyzed. The primary outcome of the study was to analyze the impact of HR on post-LT complications. A Comprehensive Complication Index ≥ 29.6 was chosen as cutoff. The secondary outcome was HCC-related death by means of competing-risk regression analysis. In the study period, 140 patients were included. Patients were transplanted in a median of 23 months after HR (IQR 14–41). Among all the features analyzed regarding the prior HR, only time interval between HR and SLT (time HR-SLT) was an independent predictor of severe complications after LT (OR = 0.98, *p* < 0.001). According to fractional polynomial regression, the probability of severe complications increased up to 15 months after HR (43%), then slowly decreased over time (OR = 0.88, *p* < 0.001). There was no significant association between HCC-related death and time HR-SLT at the multivariable competing risks regression model (SHR, 1.06; 95% CI: 0.69–1.62, *p* = 0.796). This study showed that time HR-SLT was key in predicting complications after LT, without affecting HCC-related death.

## 1. Introduction

Surgical treatment of HCC remains a valid option when it can be offered to selected patients with preserved liver function [1,2]. Even though primary liver transplantation (PLT) has been demonstrated to offer survival rates comparable to repeat hepatic resection (HR) [3], many concerns have been raised due to the shortage of donors and to the waiting-list dropout rate, thus limiting the applicability of PLT. For this reason, the policy of many centers is to indicate LT whenever HCC recurs after HR, namely salvage LT [4]. However, LT can be offered in other circumstances following HR, such as in case of liver function deterioration or adverse histo-pathological features found on the surgical specimen (e.g., microvascular invasion, positive resection margins, low grade differentiation). All of these conditions fall under the name of secondary liver transplantation (SLT) [5]. While the safety of previous HR on the postoperative outcome has been widely described in the literature for repeat hepatectomy [6], how and to what extent HR may affect the outcomes of SLT is still unknown. In this context, the main purpose of our study was to measure the impact of HR on surgical complications after SLT. The secondary study outcome was HCC-related death using competing-risk regression analysis.

## 2. Materials and Methods

### 2.1. Study Design

Data of patients treated by SLT at two tertiary referral centers (Sant’Orsola-Malpighi Hospital, Bologna, Italy and Paul Brousse Hospital, Paris, France) between January 2000 and June 2018 were retrospectively analyzed. These two centers had a large experience in HPB/LT surgery and they were chosen not only for the high number of SLT operations performed in their center, but also because they shared a similar policy for HCC patients. According to the study design, patients undergoing re-LT, without HCC, with acute liver failure (including irreversible liver failure after HR), or receiving grafts from donors after cardiac death, living donors or combined transplants, were excluded. The study protocol conformed to the ethical guidelines of the 1975 Declaration of Helsinki (6th revision, 2008). Institutional ethical committee gave approval to perform this study.

### 2.2. Variables

Variables regarding the characteristic of HR such as type of resection (major vs. minor), minimally-invasive approach, intra-abdominal complications (IAC) after HR, time between HR and SLT (time HR-SLT) or locoregional treatment (LRT) were evaluated. Major hepatic resection was defined as the removal of three or more continuous Couinaud segments. IAC following HR included all of the abdominal complications, which were supposed to increase the complexity of LT, such as pancreatic/biliary/digestive tract fistula, fluid collections (either infected or not), ascites, and hemorrhage. In the case of repeat HR, the time interval between the last surgery and SLT was taken into account to calculate time HR-SLT. LRT included trans-arterial chemoembolization (TACE), radiofrequency ablation (RFA) or percutaneous ethanol injection (PEI) with variable number, combination and sequences of application. Donor and recipient demographic data included age, etiology of liver cirrhosis, and Model for End-stage Liver Disease (MELD) score at LT. Presence of comorbidity was defined as having at least one of the following: diabetes mellitus (DM), cardiovascular disease, renal disease or pulmonary disease. Intra- and postoperative data included cold ischemia time (CIT), intraoperative blood transfusions, length of intensive care unit (ICU) stay, biliary complications, re-operation rate, hemorrhage, primary graft non-function (PGNF), hospital stay and 90-day mortality. Surgical complications were assessed by the Comprehensive Complication Index (CCI) score at discharge [7]. A CCI ≥ 29.6 was chosen as cutoff for severe complications according to the benchmark (BM) cutoffs recently reported for LT [8]. At explant pathology, largest size and number of HCC nodules, as well as presence of microvascular invasion (mVI), were also assessed.

### 2.3. Statistical Analysis

Data were expressed in median and IQR, when appropriate. The Mann–Whitney test was used for comparison of continuous variables, whereas the Chi-squared test or Fisher’s exact test were used for comparisons of categorical variables. Univariate logistic regression analysis was performed to predict the risk of developing a CCI ≥ 29.6. After evaluation of multicollinearity, multivariate logistic regression analysis was carried out on the variables, which reached *p* < 0.1 at univariate analysis. Odd ratios (OR) were adjusted for clustering on each center. All statistical tests were two-tailed, and differences were considered significant at a *p*-value of ≤0.05. Data analysis was performed with STATA for Windows (version 14).

#### 2.3.1. Fractional Polynomial Regression

Since a nonlinear relationship between time HR-SLT and the development of complications after LT was suspected, data were fitted using fractional polynomial (FP) regression techniques. The analytic approach was based on FP as developed by Royston and coworkers [9]. A CCI ≥ 29.6 was used as dependent variable and time HR-SLT as the explanatory variable. Specifically, in fitting these functions, both first-and second-degree functions were considered. A selection procedure was then used to select the best FP function (i.e., the one with the lowest deviance). Using the selected FP function, predicted values at various time points were generated. Multivariate logistic regression analysis was repeated using the polynomial function of time HR-SLT.

#### 2.3.2. Survival Analysis

Survival rates were estimated using the Kaplan–Meier method and compared using the log-rank test. In order to discriminate among deaths caused by tumor recurrence or related to other conditions (i.e., recurrence of viral hepatitis), a competing-risk analysis was implemented to define the risk of death due to HCC recurrence. The failure event was represented by “HCC-related death”, whereas “death from causes other than HCC” represented the competing event and was aimed at obtaining a sub-hazard ratio (SHR) for the prediction of the risk of death due to HCC recurrence.

## 3. Results

According to the inclusion criteria, 140 patients were analyzed. Table 1 shows the characteristics of patients at the time of HR.

Major hepatectomy was performed in 12.9% of patients (*n* = 18), whereas minimally invasive HR was performed in 15% of cases (*n* = 21). Before LT, HCC recurrence was documented in 104 patients (74.3%). Among them, 8 patients (5.7%) received a repeat HR, which included 2 further wedge resections, 2 segmentectomies, 3 bisegmentectomies and 1 right trisectionectomy. Eighty-five patients (60.7%) underwent LRTs after HR including TACE (*n* = 63, 45%), RFA (*n* = 48, 34.3%) or PEI (*n* = 3, 2.1%).

After a median of 23 months (IQR 14–41), all patients were submitted to SLT. Among them, SLT was proposed as “salvage” therapy in 90 patients (64.3%), 21 patients (15%) underwent HR as a bridge to LT, 16 patients (11.4%) were transplanted for worsening of liver function without recurrence, and 13 patients (9.3%) were deliberately (“de principe”) enlisted for LT due to the high risk of recurrence (Figure S1) [5]. Characteristics at the time of SLT are shown in Table 2.

According to the 25th, 50th and 75th percentiles, 4 subgroups were defined: (1) very early SLT (≤14 months), (2) early SLT (15–23 months), (3) late SLT (24–41 months) and (4) very late SLT (>41 months). When the patients who did not develop severe complications after SLT (CCI < 29.6, *n* = 90) were compared to those with a CCI ≥ 29.6 (*n* = 50, 35.7%) (Table 3), these two groups showed significant differences in terms of median MELD score (9 vs. 10 points, *p* = 0.005), ICU stay (4 vs. 9 days, *p* < 0.001), postoperative hemorrhage (0 vs. 12%, *p* < 0.001), re-operation rate (0 vs. 40%, *p* = 0.001), PGNF (0 vs. 10%, *p* = 0.002), re-LT (0 vs. 12%, *p* = 0.001) early biliary complications (5.5% vs. 18%, *p* = 0.019) and hospital stay (13 vs. 21 days, *p* = 0.001). Time HR-SLT was close to the margin of significance (*p* = 0.055) with a trend for a higher rate of complications in the “early SLT” group.

**Figure 1:**
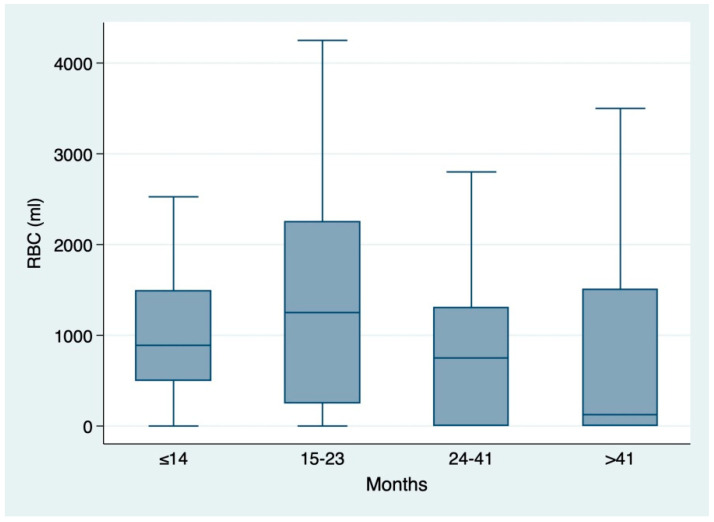
Box and whiskers plot showing the distribution of red blood counts (RBC, mL) according to the time between hepatic resection and secondary liver transplantation (time HR-SLT): ≤14 mo. (median 1000 mL, IQR 500–2250); 15–23 mo. (1250 mL, IQR 250–2500); 24–41 mo. (750 mL, IQR 0–1600); >41 (350 mL, IQR 0–1750).

A similar trend was also observed for blood transfusions when stratifying for each period of time HR-SLT (Figure 1).

**Figure 2:**
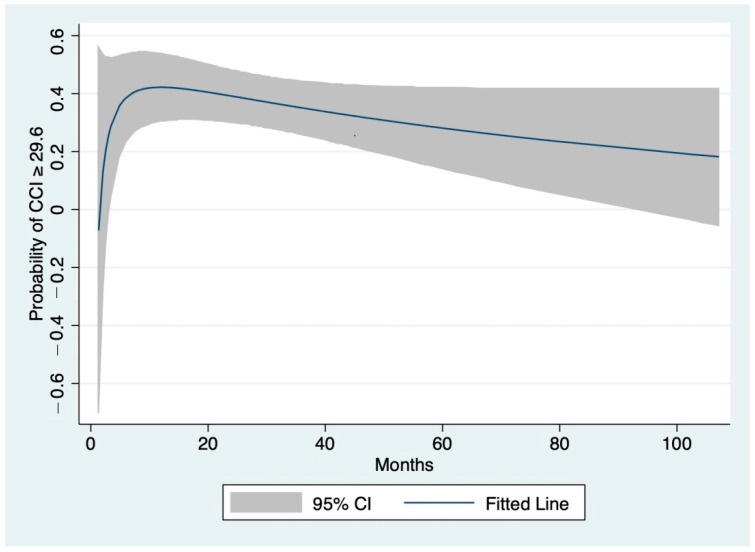
Relationship of the time between hepatic resection and secondary liver transplantation (time HR-SLT) with the probability of severe complications after SLT (Comprehensive Complication Index ≥ 29.6). The line was fitted using fractional polynomial regression and 95% confidence intervals are shown (gray shading).

The nonlinear relationship between time HR-SLT and the probability of CCI ≥ 29.6, according to FP regression, is graphically presented in Figure 2.

The probability of severe complications rapidly increased from the first measurement up to 15 months after HR (43%) before slowly decreasing at 41 months (33%).

### 3.1. Predictors of Comprehensive Complication Index (CCI) ≥ 29.6

Among all preoperative variables evaluated, including several features regarding the previous HR, univariate logistic regression analysis showed that time HR-SLT, body mass index (BMI), male sex and MELD score significantly correlated with a CCI ≥ 29.6 (Table 4). At the multivariate logistic analysis, time HR-SLT (OR = 0.98, 95% CI = 0.98–0.99, *p* < 0.001), BMI (OR = 1.07, 95% CI = 01.07–1.08, *p* < 0.001) and MELD score (OR = 1.07, 95% CI = 1.00–1.14, *p* = 0.041) were independently associated with CCI ≥ 29.6 (Table 4).

When multivariate logistic regression analysis was repeated using the polynomial function of time HR-SLT, time HR-SLT^FP^ remained significantly associated to a CCI ≥ 29.6 with a lower OR (0.88, 95% CI: 0.84–0.94, *p* < 0.001) (Table 5).

### 3.2. Survival Analysis

**Figure 3:**
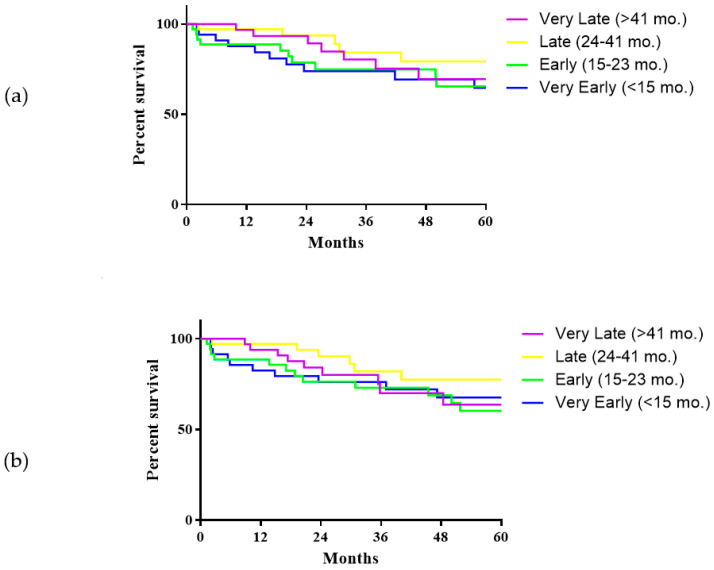
Overall survival (**a**) and recurrence-free survival (**b**) of patients submitted to secondary liver transplantation according to the time between hepatic resection and secondary liver transplantation. Overall survival: Very Early SLT vs. Early, *p* = 0.923; Very Early vs. Late, *p* = 0.128; Very Early vs. Very Late, *p* = 0.429; Early vs. Late, *p* = 0.192; Early vs. Very Late, *p* = 0.520; Late vs. Very Late, *p* = 0.510. Recurrence-free survival: Very Early SLT vs. Early, *p* = 0.717; Very Early vs. Late, *p* = 0.270; Very Early vs. Very Late, *p* = 0.929; Early vs. Late, *p* = 0.166; Early vs. Very Late, *p* = 0.880; Late vs. Very Late, *p* = 0.199.

Median follow-up was 31.8 months (IQR 17.1–78). At latest follow-up, 38 patients (27.1%) had died after SLT, of whom 19 (50%) died due to HCC recurrence. One-, 3- and 5-year overall survival (OS) rates were 92.6%, 78.6% and 69.7%. One-, 3- and 5-years recurrence-free survival (RFS) was 89.8%, 75.8% and 67.3%. When comparing the four subgroups according to time HR-SLT, no significant differences were found in this regard (Figure 3a,b).

The effect of time HR-SLT was further evaluated by the Fine and Grey’s proportional sub-distribution hazard model [10]. The variables used in the model were those that were known to have a prognostic impact after LT for HCC: number of tumor nodules, size, last AFP value available before LT and the presence of microscopic vascular invasion (mVI). Results from the competing-risk regression model are summarized in Table 6.

There was no significant association between HCC-related death and time HR-SLT in the multivariable competing risks regression model (SHR, 1.05; 95% CI: 0.68–1.62, *p* = 0.796). Largest tumor diameter (SHR, 1.05; 95% CI: 1.02–1.07, *p* = 0.001) and mVI (SHR, 4.22; 95% CI: 1.42–12.52, *p* = 0.009) were the only variables significantly associated with HCC-related death.

## 4. Discussion

This study showed that among all the features analyzed regarding the previous HR, only time HR-SLT was significantly associated with a high CCI. In particular, a longer time between HR and LT seemed to be protective against complications occurring after SLT.

According to the Barcelona Clinic Liver Cancer (BCLC) algorithm, patients with a single, very early- or early-stage HCC and preserved liver function should be considered for HR [11]. However, even after potentially curative resections with negative margins, early recurrence accounts for more than 70% and occurs within 2 years in 30–50% of patients. Repeat liver resection is often challenging due to the adhesions found at the time of second (or even third) surgery [12] and is not always feasible. In this regard, SLT can be taken into account in the case of transplantable HCC recurrence, but also in the case of worsening liver function or adverse histopathological criteria. However, Adam et al. [13] showed that SLT was associated with higher operative mortality (28.6% vs. 2.1%) compared to primary LT (PLT). Similarly, a recent meta-analysis demonstrated a significantly higher rate of postoperative bleeding and operative mortality for SLT [14]. These results are not surprising given that previous liver surgery and adhesions may impair hepatectomy during LT, thus increasing operative time and blood loss [13,15]. From the oncological point of view, when LT is performed as a “salvage” treatment, SLT seems to be superior to repeat resection in terms of OS and DFS [16] but the oncological superiority of SLT over PLT is still not clear [3,17].

However, how and to what extent a prior HR may affect the outcome in SLT has never been largely investigated. Belghiti et al. [5] showed that following major resection, SLT appeared to be more difficult, reflected by a longer operative time, increased perioperative transfusions and a longer hospital stay [5]. Our study comprised one of the largest series of SLT, joining the 20-year experience of two tertiary referral centers for HPB and LT surgery, with a similar policy for HCC patients. According to our analysis, among the different characteristics analyzed regarding the prior HR, only time interval between HR and SLT was predictive of severe complications after LT, also when adjusted for LT variables such as MELD score. In particular, an increasing probability of severe complications was observed in those patients who were transplanted close to the date of HR. Likely, these patients had more intense adhesions due to the recent hepatic surgery, which may have prolonged the time of hepatectomy as well as increased the number of blood transfusions, leading to a higher degree of complications after LT.

Other features were evaluated by means of uni- and multivariate analysis, such as major hepatic resection or IAC, which were expected to negatively affect LT outcomes due to the formation of more intense peritoneal adhesions, but they did not. BMI was found to be associated with a high CCI, although we are aware that it is not always reliable in cirrhotic patients, due to the presence of ascites [18]. The CCI summarizes all postoperative complications and has been demonstrated to be more sensitive than the existing morbidity endpoints [7]. We decided to use the cutoff of 29.6 as recently described in benchmark (BM) for LT. BM represents the best possible outcome, and the gap between BM and performance reflects the potential to improve care for individual centers [19]. Although nearly 10% of patients submitted to SLT had a pre-transplant MELD score > 20, representing a higher risk sub-group [8], major complications, biliary complications, CCI, and in-hospital mortality remained inside the BM (Table S1). This could be explained by the fact that tertiary referral centers with consistent experience in HPB surgery and LT may have benefits in both fields, especially when these fields are related to each other [20,21]. Only ICU stay and operation duration were outside the BM, the latter likely due to a longer time spent on adhesiolysis, normally occurring in patients with prior upper abdominal surgery [22,23,24].

With regard to the oncologic outcomes, a longer time between HR and SLT was found to not affect HCC-related death, and instead was associated with tumor diameter and presence of mVI, confirming that tumor characteristics prevailed over timing of SLT [25,26]. Even though there could be a potentially increased risk of recurrence while waiting for LT, we believe that a minimum time before LT could be helpful in decreasing complications after LT as well as in selecting patients based on the biological aggressiveness of disease. However, this study was not conducted on an intention-to-treat basis and dropout rate while time on the waiting list was not considered, thus limiting our conclusions. Furthermore, the response to LRTs, which is increasingly being recognized as one of the most important determinants of HCC recurrence after LT [27,28] was not available for every patient, meaning it was not possible to adjust the analysis for this variable. In addition, the question of whether a laparoscopic approach might have improved these specific outcomes was not addressed in this study. Although minimally-invasive liver resection has been demonstrated to facilitate subsequent LT in terms of blood loss and transfusion requirements [29,30,31], we were not able to confirm the hypothesis that a laparoscopic approach may improve short-term outcomes post-SLT. Again, the center experience in both HPB surgery and LT could have contributed to minimizing the difference in post-LT results. Nevertheless, we acknowledge that this important aspect surely requires further dedicated studies [32,33,34].

## 5. Conclusions

In conclusion, this study showed that the time interval between HR and SLT was key in predicting complications after SLT, without affecting HCC-related death. Multicenter trials are warranted to confirm the potential advantages of a laparoscopic approach compared to open resection to improve these specific outcomes.

## Figures and Tables

**Table 1:** Characteristics of patients at the time of hepatic resection (HR).

Variable	*n* = 140
Age, median (range), years	56 (12–70)
LRT before HR, *n* (%)	27 (19.3)
Laparoscopic approach, *n* (%)	21 (15)
Type of HR, *n* (%)	
Wedge	44 (31.4)
Bisegmentectomy	39 (27.9)
Segmentectomy	39 (27.8)
Right hepatectomy	9 (6.4)
Left hepatectomy	6 (4.3)
Right trisectionectomy	2 (1.4)
Central hepatectomy	1 (0.8)
HR +RFA, *n* (%)	9 (6.4)
IAC after HR, *n* (%)	27 (19)
Tumor number, median (IQR), *n*	1 (1–2)
Tumor diameter max, median (IQR), mm	29 (20–44)

HCC = hepatocellular carcinoma; HR = hepatic resection; IAC = intra-abdominal complications; LRT = locoregional treatment; RFA = radiofrequency ablation.

**Table 2:** Characteristics of the patients at the time of secondary liver transplantation (SLT).

Variable	*n* = 140
Age, median (range), years	59 (12–73)
Male sex, *n* (%)	123 (88)
Cardiovascular disease, *n* (%)	51 (36.4)
Renal disease, *n* (%)	16 (11.4)
Pulmonary disease, *n* (%)	33 (23.6)
Diabetes, *n* (%)	41 (29.3)
BMI, median, (IQR), kg/m^2^	26 (23–28)
MELD, median, (IQR), points	9 (8–12)
MELD > 20, *n* (%)	14 (10)
Viral etiology, *n* (%)	97 (70)
Time HR-SLT, median (IQR) months	23.1 (IQR: 14–41)
SLT era, *n* (%) 2000–2009 2010–2018	45 (32) 95 (68)
Donor age, median (range), years	59 (10–87)
Tumor number, median (IQR), *n* *	1 (0–3)
Tumor diameter max, median (IQR), mm *	13 (0–23)
mVI+, *n* (%) *	48 (34.5)
Alpha-fetoprotein, median (IQR), ng/mL	7 (4–17)
Cold Ischemia Time, median (IQR), min	407 (339–510)
Operative Time, median (IQR), min	480 (405–600)
Blood transfusion, median (IQR), ml	889 (0–2250)
ICU stay, median (IQR), days	5 (3–8)
Post-operative hemorrhage, *n* (%)	6 (4.3)
Biliary complications, *n* (%)	14 (10)
Hepatic artery thrombosis, *n* (%)	1 (0.7)
Re-operation, *n* (%)	20 (14.3)
PGNF, *n* (%)	5 (3.6)
Re-LT, *n* (%)	6 (4.3)
CCI, median (IQR), points	20.9 (0–42.4)
Clavien ≥ IIIA morbidity, *n* (%)	49 (35)
Hospital stay, median (IQR), days	16 (0–25)
In-hospital mortality, *n* (%)	2 (1.4)
90-day mortality, *n* (%)	7 (5)

BMI = body mass index; CCI = Comprehensive Complication Index; IQR = interquartile range; HR = hepatic resection; ICU = intensive care unit; LRT = locoregional treatment; MELD = Model for End-Stage Liver Disease; mVI = microvascular invasion; PGNF = primary graft non- function; SLT = secondary liver transplantation; * at explant pathology.

**Table 3:** Characteristics of patients with or without severe complications (Comprehensive Complication Index CCI ≥ 29.6).

Variable	CCI < 29.6 (*n* = 90)	CCI ≥ 29.6 (*n* = 50)	*p*-Value
Male sex, *n* (%)	76 (84)	47 (94)	0.097
BMI, median (IQR), kg/m^2^	25.1 (23–27)	27 (24–29)	0.041
Comorbidities, *n* (%)	32 (35)	25 (50)	0.096
LRT after HR, *n* (%)	60 (67)	34 (68)	0.872
SLT era (2010–2018), *n* (%)	56 (62)	39 (78)	0.055
Laparoscopic approach, *n* (%)	13 (14)	8 (16)	0.805
Type of HR			0.775
Segmentectomy, *n* (%)	27 (30)	12 (24)	
Wedge, *n* (%)	26 (29)	18 (36)	
Bisegmentectomy, *n* (%)	26 (29)	13 (26)	
Major hepatectomy, *n* (%)	11 (12)	7 (14)	
HR + RFA, *n* (%)	6 (7)	3 (6)	0.878
Repeat HR	4 (4.4)	4 (8)	0.385
IAC after HR, *n* (%)	50 (56)	28 (56)	0.960
Time HR-SLT, *n* (%)			
≤14 mo.	25 (71.4)	10 (28.6)	0.055
15–23 mo.	16 (45.7)	19 (54.3)	
24–41 mo.	23 (65.7)	12 (34.3)	
>41 mo.	26 (74.3)	9 (25.7)	
Recipient age, median (range), years	58 (16–73)	60 (12–70)	0.429
MELD, median, (IQR), points	9 (7–12)	10 (9–15)	0.005
Donor age, median (range), years	58 (17–87)	63 (10–85)	0.155
ICU stay, median (IQR), days	4 (3–6)	9 (6–13)	<0.001
Blood transfusion, median (IQR), ml	642 (0–1735)	1058 (500–2400)	0.069
Biliary complications, *n* (%)	5 (5.5)	9 (18)	0.019
Hepatic artery thrombosis, *n* (%)	0 (0)	1 (2)	0.178
Re-operation, *n* (%)	0 (0)	20 (40)	<0.001
Post-operative hemorrhage, *n* (%)	0 (0)	6 (12)	0.001
Hospital stay, median (IQR), days	13 (9–20)	21 (15–29)	0.001
PGNF, *n* (%)	0	5 (10)	0.002
Re-LT, *n* (%)	0 (0)	6 (12)	0.001
90-day mortality, *n* (%)	3 (3)	4 (8)	0.225

BMI = body mass index; CCI = Comprehensive Complication Index; HR = hepatic resection; IAC = intra-abdominal complications; IQR = interquartile range; ICU = intensive care unit; LRT = locoregional treatment; MELD = Model for End-Stage Liver Disease; PGNF = primary graft non-function; RFA = radiofrequency ablation; SLT = secondary liver transplantation.

**Table 4:** Uni-and multivariate analysis of predictors of severe complications after SLT (Comprehensive Complication Index ≥ 29.6).

Variable	Univariate			Multivariate		
	OR	95% CI	*p*-Value	OR	95% CI	*p*-Value
Male Sex	2.89	2.44–3.42	<0.001	4.67	0.97–22.32	0.053
Comorbidities	1.81	0.89–3.69	0.101			
BMI (per 1 kg/m^2^)	1.05	1.03–1.08	<0.001	1.07	1.07–1.08	<0.001
TACE	1.35	0.87–2.11	0.182			
RFA	0.74	0.49–1.11	0.144			
Major hepatectomy	1.17	0.75–1.83	0.494			
Repeat resection	1.87	0.75–4.67	0.181			
Laparoscopic approach	1.13	0.35–3.65	0.840			
Time HR-SLT (per 1 mo.)	0.98	0.98–0.99	0.007	0.98	0.98–0.99	<0.001
IAC post-HR	1.02	0.68–1.53	0.931			
SLT era (2010–2018)	2.15	0.81–5.70	0.123			
Recipient age (per 1 yr)	1.01	0.98–1.04	0.521			
MELD score (per 1 point)	1.06	1.01–1.12	0.022	1.08	0.98–1.18	0.041
Donor age > 60 yrs	1.49	0.70–3.17	0.300			

BMI = body mass index; HR = hepatic resection; IAC = intra-abdominal complications; MELD = Model for End-Stage Liver Disease; RFA = radiofrequency ablation; SLT = secondary liver transplantation; TACE = trans-arterial chemoembolization.

**Table 5:** Multivariate analysis of predictors of severe complications after SLT using fractional polynomial (FP) function of time HR-SLT.

Variable		Multivariate Analysis	
	OR	95% CI	*p*-Value
BMI	1.07	1.07–1.08	<0.001
Male Sex	4.6	0.95–22.57	0.058
MELD score	1.07	1.00–1.14	0.036
time HR-SLT^FP^	0.88	0.84–0.94	<0.001

BMI = body mass index; FP = fractional polynomial; HR = hepatic resection; MELD = Model for End-Stage Liver Disease; SLT = secondary liver transplantation.

**Table 6:** Competing risk regression analysis on cancer-related death.

Variable	Competing Risk Regression		
	sHR	CI 95%	*p*-Value
Last AFP before LT	0.99	0.99–1.00	0.351
mVI *	4.22	1.42–12.52	0.009
Tumor number *	1.02	0.97–1.06	0.504
Tumor diameter *	1.05	1.02–1.07	0.001
Time HR-SLT^FP^	1.06	0.69–1.62	0.796

AFP = alpha-fetoprotein; BMI = body mass index; FP = fractional polynomial; HR = hepatic resection; mVI = microvascular invasion; sHR = sub-hazard ratio; SLT = secondary liver transplantation; * at explant pathology.

## Data Availability

The data presented in this study are available on request from the corresponding author. The data are not publicly available due to privacy reasons.

## Supplementary Materials

The following are available online at https://www.mdpi.com/article/10.3390/cancers13102398/s1, Figure S1: Flow-chart of the study; Table S1: Comparison between benchmark cutoffs for liver transplantation (LT) and the outcomes after secondary LT.
