# Supplementary material for: The Impact of Time Interval between Hepatic Resection and Liver Transplantation on Clinical Outcome in Patients with Hepatocellular Carcinoma
Source: Cancers (Basel). 2021 May 15;13(10):2398. doi: 10.3390/cancers13102398 (PMC8156967; doi:10.3390/cancers13102398)
Supplement: Supplementary file 1 [file cancers-13-02398-s001.zip › cancers-1204085-supplementary.pdf]

cancers

MDPI

Supplementary

The Impact of Time Interval between Hepatic Resection and Liver Transplantation on Clinical Outcome in Patients with Hepatocellular Carcinoma

Matteo Serenari et. al.

Figure S1. Flow-chart of the study.

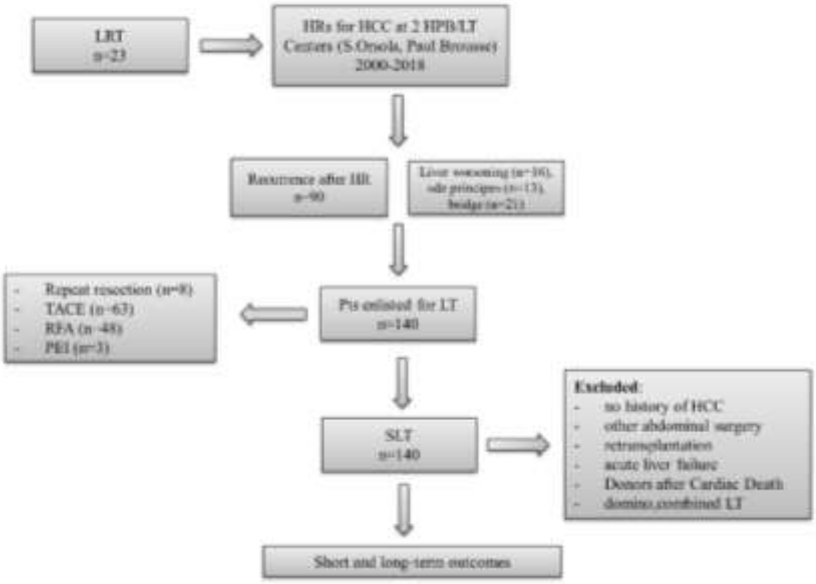

LRT = locoregional treatment; HR = hepatic resection; HCC= hepatocellular carcinoma; HPB= hepato-biliary; LT= liver transplantation; SLT= secondary liver transplantation; TACE = transarterial chemoembolization; RFA = radiofrequency ablation; PEI = percutaneous ethanol injection.

Table S1. Comparison between benchmark cutoffs for liver transplantation (LT) and the outcomes after secondary LT.

	BENCHMARK	SLT
**Perioperative Course**		
OP duration	≤6 h	8 h
Intraoperative blood transfusions	≤900 cc (3U)	889 cc
ICU stay	≤4 days	5 days
Hospital stay	≤18 days	16 days
**Morbidity and Mortality**		
Clavien ≥ IIIA morbidity	≤42%	35%
Biliary complications	≤12%	10%
Hepatic artery thrombosis	≤4.1%	0.7%
CCI	≤29.6	20.9
Graft-loss	≤4%	3.6%
Mortality	≤2%	1.4%

OP = operation; SLT = secondary liver transplantation; ICU = intensive care unit; CCI = Comprehensive Complication Index.
